# Anti‐fibrotic effect of ciglitazone in HRV‐induced airway remodelling cell model

**DOI:** 10.1111/jcmm.17790

**Published:** 2023-05-31

**Authors:** Joanna Wieczfinska, Rafal Pawliczak

**Affiliations:** ^1^ Department of Immunopathology Medical University of Lodz Lodz Poland

**Keywords:** airway remodelling, asthma, ciglitazone, collagen I, MMP‐9, PPAR‐γ, TGF‐β

## Abstract

Fibrosis is an important phenomenon as it can occur early in the pathogenesis of asthma; it may be associated with disease severity and resistance to therapy. There is a strong evidence that infection caused by human rhinovirus (HRV) contributes to remodelling process, but there is lack of studies clearly explaining this pathway. Synthetic peroxisome proliferator‐activated receptor (PPAR) γ presents immunomodulatory and anti‐inflammatory features. In this study, we examined immunomodulatory properties of ciglitazone – PPAR‐γ agonist, in development and modulation of airway remodelling. Epithelial cells (NHBE) and two lines of fibroblasts (WI‐38, HFL1) were stimulated with ciglitazone and rhinovirus. The expression of genes related to airway remodelling process were analysed in the cells; moreover NF‐κB, c‐Myc and STAT3 were silenced in order to estimate potential pathways involved. Ciglitazone decreased mRNA expression of MMP‐9 and TGF‐β. It also modified the expression of α‐SMA and collagen after rhinovirus infection. Transcription factors knockdown altered the levels of expression. The results suggest possible anti‐fibrotic activity of PPAR‐γ agonist in human airway cells. Ciglitazone has been shown to be dependent on NF‐κB‐ and STAT3‐related pathways, thus, the PPAR‐γ agonist may have therapeutic potential for the treatment of airway remodelling in asthma.

## 1 INTRODUCTION

Bronchial asthma is a common chronic inflammatory disorder of the airways, caused by immune and inflammatory responses and is characterized by reversible airflow obstruction.^1^ One of the central feature of asthma is airway remodelling; defined as changes in airway wall structure, including extensive epithelial damage, airway smooth muscle hypertrophy, collagen deposition, subepithelial basement membrane thickness and fibrosis. Asthma is an incurable nonetheless preventable disease; currently available treatments control airway inflammation and symptoms, but only to a certain degree.^2^

It has been demonstrated previously that human airway cells infected with HRV may induce a number of growth factors as well as chemotactic agents connected to airway remodelling, that can drive mesenchymal cells to migrate towards the epithelial layer.^3^ Minor et al^4^ directly demonstrated that two distinct HRV serotypes are also capable of inducing EMT‐related epithelial phenotypic and morphological alterations, further demonstrating the potential contribution of HRV infections to airway remodelling.

A number of mediators, including TGF‐β, MMP‐9, and ADAM33, have been identified to control airway remodelling.^5^ TGF‐β is a crucial profibrotic cytokine, which promotes the development of fibroblasts into myofibroblasts, that release growth factors and collagen. TGF‐β expression correlates with thickness of basement membrane, fibroblast numbers, and severity of the disease.^6^ The complicated interaction between TGF‐β1 and STAT3 appears to serve a variety of biological settings. Numerous fibrotic tissues have shown STAT3 activation; this occurs as a result of the inflammatory response, which is the initial phase of wound healing.^7^ However, the molecular processes which would clarify the role of STAT3 in the initiation and development of fibrosis are still unknown.

STAT3 as well as NF‐κB both regulate the expression of a large number of downstream genes that control immune functions, stress responses, cell proliferation and survival. Additionally, the two transcription factors are engaged in both positive and negative crosstalk.^8^ Several studies demonstrate contribution of STAT3 to fibrosis through induction of ECM (extracellular matrix) production.^9^, ^10^, ^11^ TGF‐β has a major impact on the production of the ECM via its downstream effectors and it is crucial in signalling net leading to fibrosis.^12^

NF‐κB signalling in asthma has been demonstrated by increased IκB (inhibitor of NF‐κB) phosphorylation or degradation, increased NF‐κB nuclear localization or DNA binding, and increased IκB kinase complex (IKK)‐β expression in the airway tissue.^13^ Moreover, increased NF‐κB has been also detected in induced sputum inflammatory cells of the asthmatic patients, and inhaled budesonide has been effective to decrease the levels of activated NF‐κB in human airway wall tissue.^14^

The superfamily of ligand‐activated nuclear receptors includes transcriptional factors called peroxisome proliferator‐activated receptors (PPARs). There have been recognized as three main types: PPAR‐α, PPAR‐βδ, and PPAR‐γ, and the last one is known to play a crucial role in airway inflammatory responses regulation. Previous studies demonstrated the role of PPAR‐γ in asthmatic airway remodelling and inflammation.^15^, ^16^ PPAR‐γ expression is increased in the airway mucosa of asthmatics in comparison to healthy subjects,^17^ moreover, PPAR‐γ inhibits expression of many cytokines and activation of inflammatory cells which are increased in asthma.^16^

The thiazolidinedione class of anti‐diabetic medications as well as naturally occurring ligands may both activate PPAR‐γ, which is up‐regulated by TGF‐β1. The sensitivity of fibroblasts to PPAR‐ligands is greatly increased by transient overexpression of PPAR‐γ. 15d‐PGJ2 as well as troglitazone (PPAR‐γ agonists) inhibit TGF‐β‐induced transcriptional activation of type I collagen production and abolish α‐SMA expression.^18^ The PPAR‐γ ligands have distinct and PPAR‐γ dependent inhibitory effects on these profibrotic reactions. Moreover, the degree of cellular Smad3 or Smad7 expression is unaffected by PPAR‐γ activation in fibroblasts. This might mean that PPAR‐γ prevents TGF‐β from inducing profibrotic responses in healthy fibroblasts.^18^ There is a proof that PPAR‐activation may control the ECM deposition involved in the remodelling of the airway wall. In response to antigen sensitization and challenge, ciglitazone reduced basement membrane thickness and airway collagen deposition, which was accompanied by a decrease in TGF‐β production.^19^ Ciglitazone has also been demonstrated to block TGF‐β signalling in cultured lung fibroblasts.^20^ Moreover, previous work by Chima et al. indicated that ciglitazone ameliorated lung inflammation by modulating the IKK/NF‐κB pathway which was mediated through inhibition of the IKK/NF‐κB pathway.^21^

The aim of the study was to examine the signalling mechanisms for airway remodelling development, the role of chosen transcription factors – NF‐κB, c‐myc and STAT3, and to determine the effect of PPAR‐γ agonist – ciglitazone influence on the expression of airway remodelling‐related genes in the presence of rhinoviruses (HRV‐2 and HRV‐16) infections – factors triggering airway remodelling changes.

## 2 MATERIALS AND METHODS

### 2.1 Cell cultures

WI‐38 and HFL1 fibroblasts were purchased from Sigma‐Aldrich and cultured in EMEM medium (WI‐38) and HAM's12 medium (HFL1) with 10% fetal bovine serum, 2 mM of L‐glutamine, 1% of non‐essential amino acids and standard Penicillin Streptomycin solution (Sigma‐Aldrich). NHBE epithelial cell line was purchased from Lonza (Lonza Walkersville Inc.) and grown in Bronchial Epithelial Cell Growth Medium BulletKit (Lonza Walkersville Inc.). The experiments (*n* = 6), were performed after reaching 80%–90% confluence by the cells. The viability of the cells was assessed by adding 10 μL of Presto Blue (BD Pharmingen) and the absorbance was measured at 570 nm.

### 2.2 Virus preparation and cell infection

Two serotypes of human rhinoviruses (HRV) 16 and HRV‐2 were purchased from the European Collection of Authenticated Cell Cultures (ECACC)). Ohio HeLa cell line, purchased from Sigma‐Aldrich was infected until cytopathic effect was observed multiplicity of infection (MOI) of 1, established on the base of literature.^22^, ^23^ HRV specimens were exposed to the temperature of 58°C for 1 h in order to inactivate the virus particles,^24^ which was subsequently confirmed by a lack of HRV replication.

The target fibroblast and epithelial cells were infected by the addition of 50 μL vehicle (medium) or HRV16. The cells were incubated for 24 h (33°C, 5% CO_2_).^25^

### 2.3 Experimental procedure

The cultures were exposed to the HRV‐2 (minor serotype) and HRV‐16 (major serotype) virus for 24 h (33°C, 5% CO_2_). Following this, the cells were incubated with ciglitazone (3 μM) for 24 h (37°C, 5% CO_2_). Some of the wells were treated the other way – first ciglitazone was added (3 μM) for 24 h (37°C, 5% CO_2_), and then, the cells were infected by HRV‐2 or HRV‐16. The aim of such action was to investigate whether there were differences at the molecular level due to the order in which ciglitazone was added. Namely, whether ciglitazone can prevent HRV‐induced changes if it is added before the infection, or whether it can inhibit HRV action when it added after cells were infected. Ciglitazone added to cells before infection may have a preventive effect on the changes subsequently induced by HRV. On the other hand, ciglitazone added to already infected cells may have a mitigating effect; therefore, one of our goals was to analyse the effect of the order in which ciglitazone was added to cells in the context of HRV infection.

The controls contained medium only. We have chosen one HRV serotype from each group in order to evaluate potential differences between them. All the experiments were performed three times in duplicate (passages 3 to 9). The time‐point chosen by out of three (6, 12 and 24 h, data not shown), consistent with the literature.

### 2.4 RNA isolation and cDNA synthesis

Total RNA was isolated from the cells by using a Total RNA mini kit (A&A Biotechnology). The RNA was next purified and stored at −80°C. Reverse transcription was performed using a High Capacity cDNA kit (Applied Biosystems), using 1 μg of total RNA. The procedures were performed according to the producer's protocols.

### 2.5 Gene expression analysis

The changes in the expression of metalloproteinase ‐9, transforming growth factor β1(TGF‐β1), collagen I, disintegrin and metalloproteinase domain‐containing protein 33 (ADAM33), chitinase‐3‐like protein 1 (YKL‐40), relaxin/insulin‐like family peptide receptor 1 (RXFP1), leukotriene C4 synthase (LTC4S) and alpha‐SM‐actin (α‐SMA) were assessed utilizing qPCR. TaqMan gene expression assays were used for the selected genes: MMP‐9 – Hs00957562_m1, TGF‐β1 – Hs00998133_m1, collagen I – Hs00164004_m1, ADAM33 – Hs00905552_m1, YKL‐40 – Hs01072228_m1, RXFP1 – Hs01073145_m1, LTC4S – Hs01073145_m1, α‐SMA – Hs05005339_m1, and β‐actin – Hs99999903_m1 (Life Technologies). Each sample was measured in triplicate using the TaqMan analyser and the 2^−ΔΔCt^ method was used to calculate gene expression. The results were normalized to an endogenous reference gene (β‐actin – Hs99999903_m1). LTC4 synthase was evaluated as an inflammation marker and α‐SMA – as fibroblast‐myofibroblasts transformation marker. By comparing RQ (relative quantification, 2^−ΔΔCt^), the fold change in mRNA expression was calculated.

### 2.6 Determination of protein concentrations

Commercially enzyme‐linked immunosorbent assay (ELISA) kits were used to measure MMP‐9 (ab100610), TGF‐β1 (ab100647), Collagen I (ab285250), YKL‐40 (ab255719), α‐SMA (ab240678, Abcam) and ADAM33 (MBS8805359), RXFP1 (MBS9337101) and LTC4S (MBS2602658, MyBioSource, Inc.) in the supernatant of cell cultures in triplicate, according to the manufacturer's instructions. PPAR‐γ was determined by Human PPAR‐gamma ELISA Kit (MyBioSource, Inc.).

### 2.7 siRNA silencing of transcription factors

For the knockdown of selected genes (NF‐κB, c‐Myc and STAT3), a Silencer siRNA Transfection Kit was used, according to the manufacturer's instructions (Thermo Fisher Scientific). The cells were treated with 20 nM siRNA mixture against NF‐κB (NCBI accession no. NM_001145138.1), c‐Myc (NCBI accession no. NM_002467.4) and STAT3 mRNA (NCBI accession no. NM_003150.3), (Thermo Fisher Scientific) for 48 h. The same concentration of scrambled siRNAs was used as the negative control. The knockdown efficiency was evaluated after 48 h of transfection. More than 80% decorin silencing/knockdown was achieved as confirmed by real time PCR.

### 2.8 Statistical analyses

The obtained results were analysed with software Statistica software (StatSoft). The Shapiro–Wilk test and Levene's test, were respectively utilized to check the distribution of data as well as the equality of variances. Significant changes were calculated using the anova test with the appropriate post hoc tests as a multiple comparison procedure. *p* < 0.05 were considered to be statistically significant.

## 3 RESULTS

### 3.1 Ciglitazone effect on airway remodelling‐related genes expression

**FIGURE 1:**
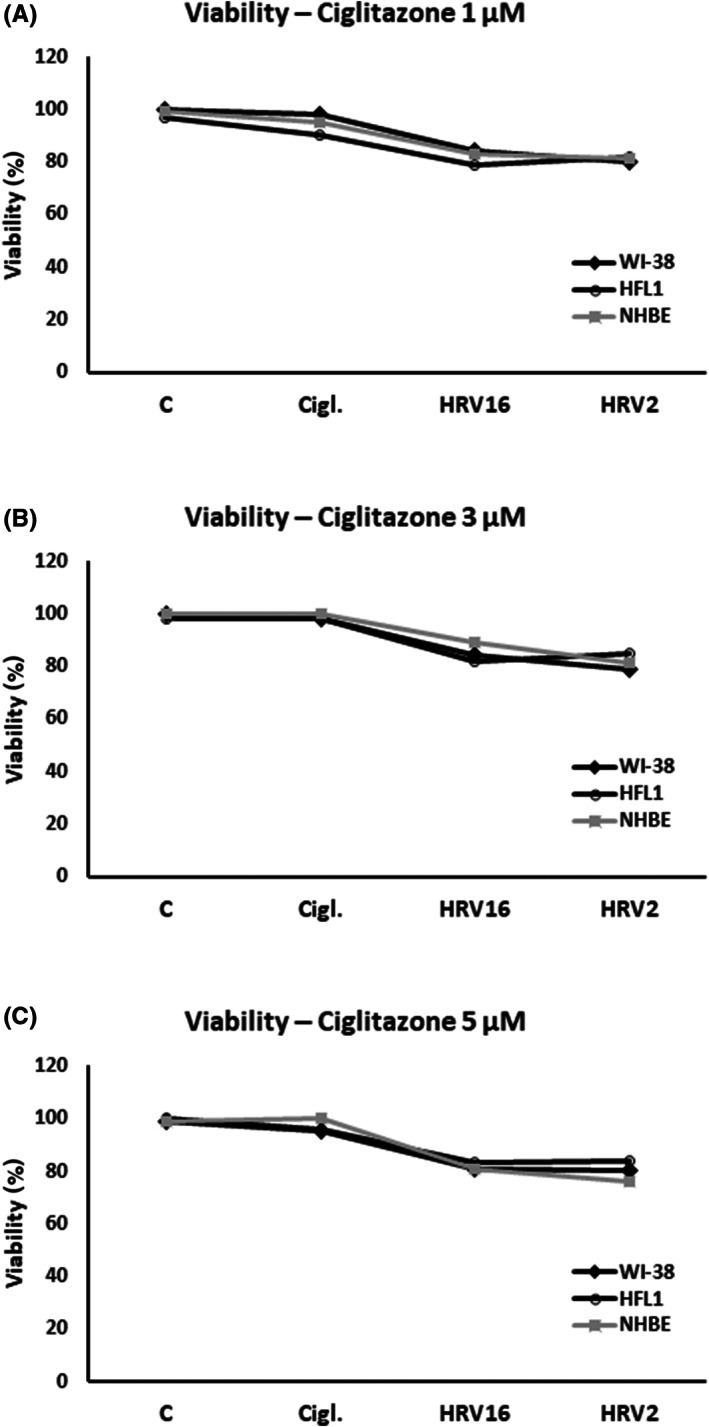
Viability of WI‐38, HFL1 and NHBE cells after exposure to ciglitazone in concentration of 1 μM (A), 3 μM (B) and 5 μM (C). Cell viability was assessed after 24 h. The values represent mean viability.

**FIGURE 2:**
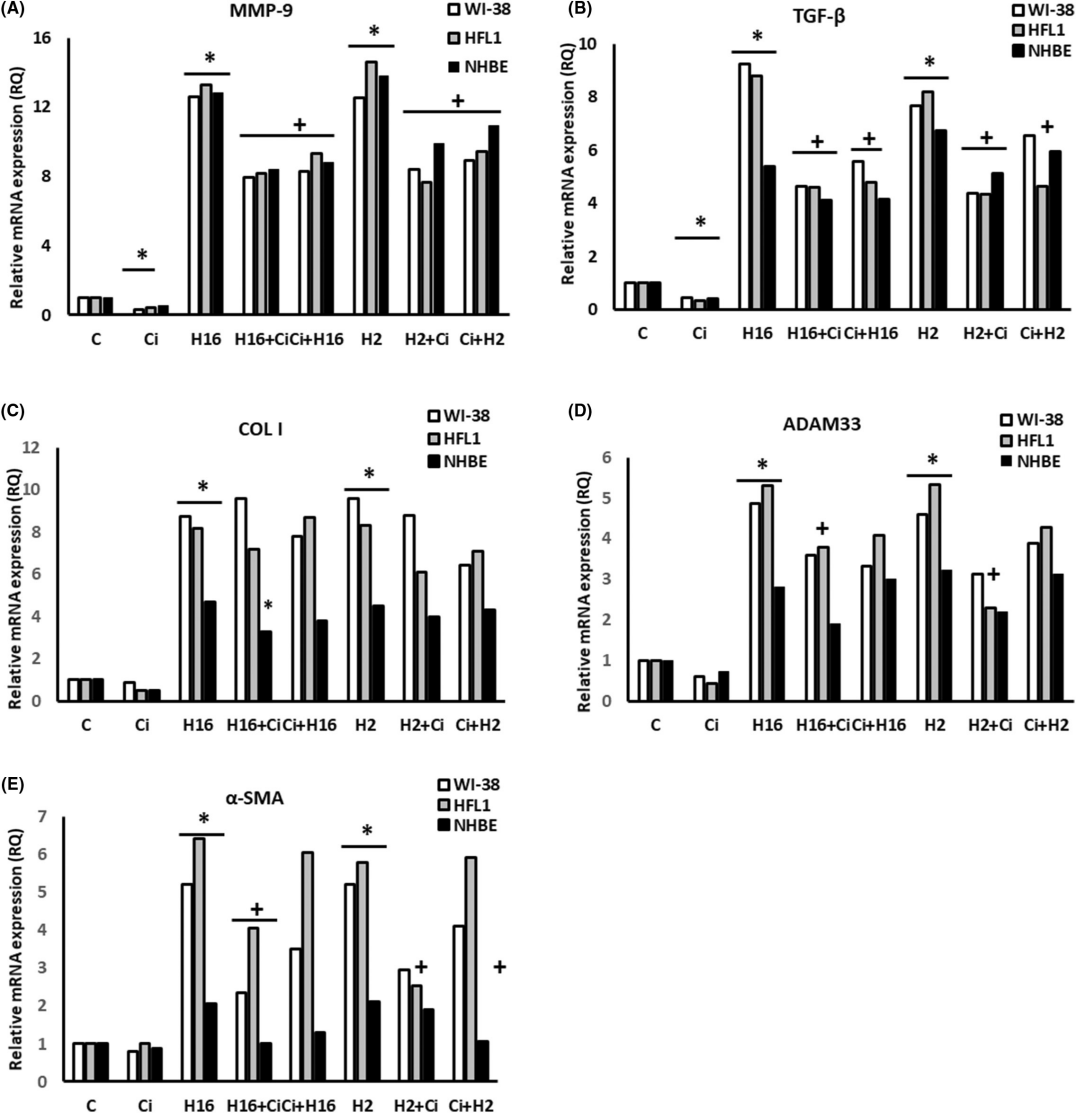
The result of ciglitazone stimulation on rhinovirus‐triggered mRNA expression of airway remodelling‐involved genes. Two serotypes of Rhinovirus 2 (minor serotype) and 16 (major serotype) were used. Ciglitazone (Ci) has been added before rhinovirus infection (Ci+H16, Ci+H2) or after rhinovirus infection (H16+Ci, H2+Ci). Ciglitazone decreased the expression of MMP‐9 (A) in fibroblasts, and TGF‐β (B) in fibroblasts and epithelial cells (*p* < 0.05). Rhinoviruses increased the expression of all genes analysed in fibroblasts (*p* < 0.05). Ciglitazone reversed the effect of rhinoviruses by decreasing the mRNA expression of MMP‐9 (A), TGF‐β (B), ADAM33 (D), and α‐SMA (E) in fibroblasts, whereas in the case of epithelial cells, its action was observed in the COL I expression only (C, *p* < 0.05). *p < 0.05 in comparison to the control sample, +*p* < 0.05 in comparison to the rhinovirus sample (HRV‐2 or HRV‐16, respectively); C – control sample with medium only.

Ciglitazone showed no cytotoxicity towards any of the cell lines used (Figure 1). The effect of ciglitazone caused noticeable changes in MMP‐9 and TGF‐β1 mRNA expressions, resulting in reduced rhinovirus‐induced mRNA expression (HRV‐16 and HRV‐2), for all cell types tested (Figure 2A,B). For example, for WI‐38, RQ of MMP‐9 was reduced from 12.61 (HRV‐16) to 7.93 (HRV‐16+ciglitazone), and 8.31 (ciglitazone+HRV‐16) (*p* < 0.05, Figure 2A). In all cell lines used, collagen I mRNA expression did not change significantly after stimulation with ciglitazone alone; however, in epithelial cells, ciglitazone reduced HRV‐16‐induced mRNA expression of this gene (RQ = 4.7 vs. 3.3), but only the experimental system when ciglitazone was added to HRV‐16 infected cells, not vice versa (Figure 2C).

Similar results were obtained when mRNA expression of ADAM33 and α‐SMA was examined – ciglitazone added to cells did not induce statistically significant changes, but reduced HRV‐induced (both serotypes) mRNA expression of the above genes (Figure 2D,E). However, for ADAM33, this effect was only observed in fibroblast cells (for example, RQ for HFL1 = 5.29 after HRV‐16 vs. 3.79 after HRV‐16+ciglitazone (*p* < 0.05)), with the effect not observed when ciglitazone was added prior infection (Figure 2D).

**FIGURE 3:**
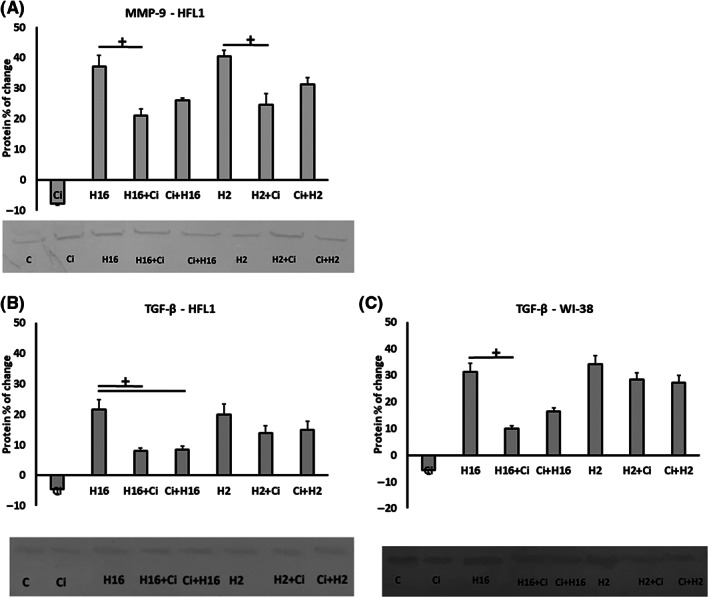
Effect of ciglitazone to reverse HRV‐stimulated proteins. Fibroblasts (A, C – HFL1 cell line and B – WI‐38 cell line) were infected with human rhinovirus and stimulated with PPAR‐γ agonist – ciglitazone (Ci). The cells were chosen to be presented, as no changes were observed in epithelial cells. Ciglitazone (Ci) has been added before rhinovirus infection (Ci+H16, Ci+H2) or after rhinovirus infection (H16+Ci, H2+Ci). Concentrations of MMP‐9 (A) and TGF‐β (B,C), were measured in supernatants utilizing immunoassay in triplicate. Data presented as % of change, normalizing to control sample ± SD. +<0.05. *p < 0.05 in comparison to the control sample, +*p* < 0.05 in comparison to the rhinovirus sample (HRV‐2 or HRV‐16, respectively).

Protein expression analysis showed statistical significance for HFL1 cells – ciglitazone decreased HRV‐2 and HRV‐16 induced MMP‐9 protein expression (*p* < 0.05, Figure 3A). In addition, statistical significance was observed for TGF‐β1 protein expression – for HFL1 cells (Figure 3B), ciglitazone decreased HRV‐16‐induced protein expression; both in the experimental system when ciglitazone was added to the existing infection and when it was added before the infection (*p* < 0.05), while for WI‐38 cells – a significant change appeared only when ciglitazone was added to the existing infection (*p* < 0.05, Figure 3C). The other expression values of the tested proteins showed no statistical significance (*p* > 0.05).

### 3.2 Transcription factors knockdown

#### 3.2.1 NF‐κB

**FIGURE 4:**
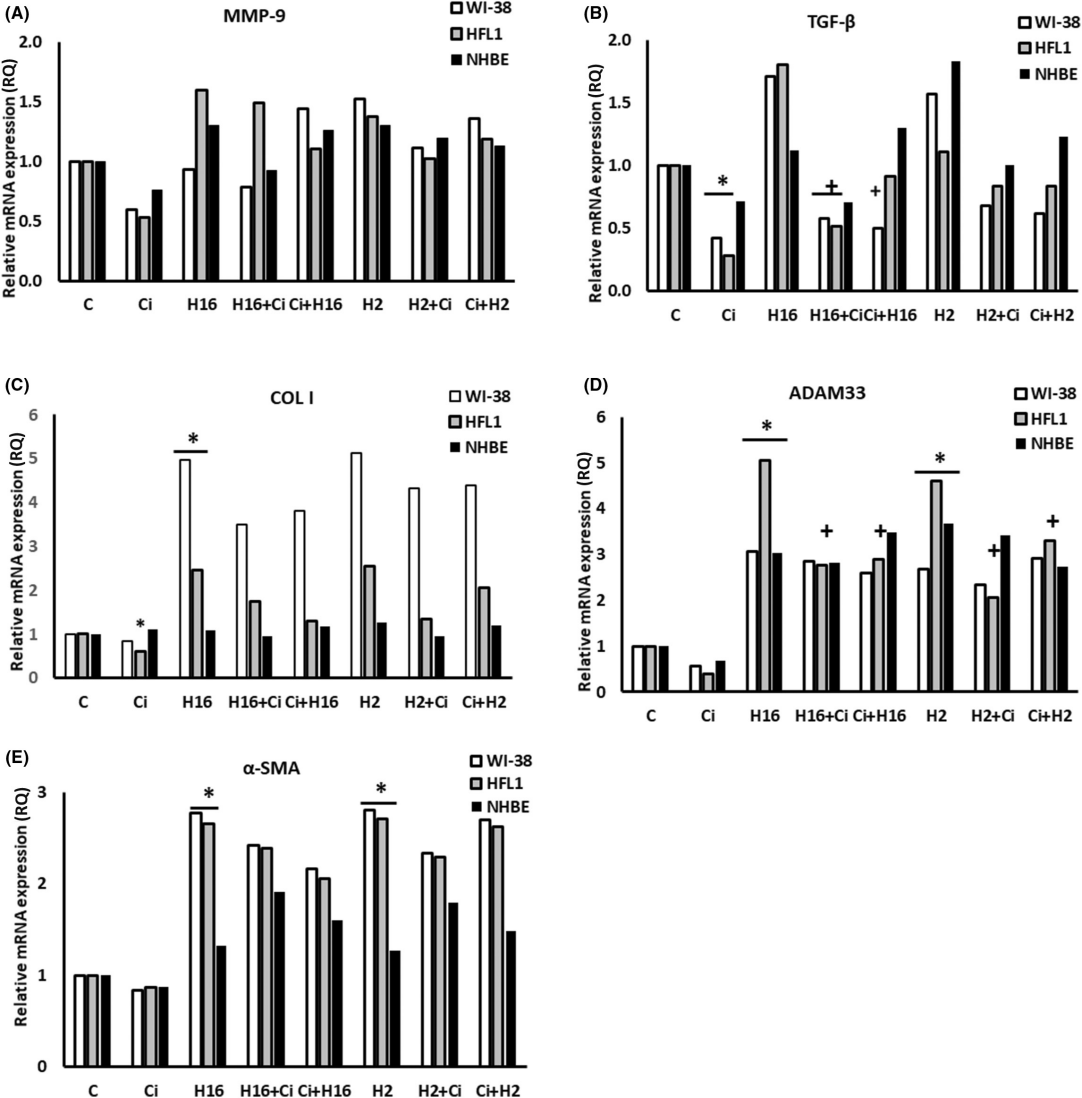
The effect of ciglitazone treatment on remodelling process induced by rhinovirus, under conditions of siRNA knock down of NF‐κB transcription factor. Rhinovirus 2 (H2, minor serotype) and 16 (H16, major serotype) were used. Ciglitazone (Ci) has been added before rhinovirus infection (Ci+H16, Ci+H2) or after rhinovirus infection (H16+Ci, H2+Ci). NF‐κB silencing eliminated effects of HRV and ciglitazone in MMP‐9 (A) and TGF‐β (B), (*p* > 0.05). The knockdown reversed changes introduced by ciglitazone in the expression of MMP‐9 (A), COL I (C), α‐SMA (E), and partly in TGF‐β (B) and ADAM 33 (D), in comparison to cells expressing NF‐κB (*p* < 0.05). *p < 0.05 in comparison to the control sample, +*p* < 0.05 in comparison to the rhinovirus sample (HRV‐2 or HRV‐16, respectively); C – control sample with medium only.

After silencing of the transcription factor NF‐κB, no changes were observed in the mRNA expression levels of MMP‐9 (*p* > 0.05, Figure 4A), also none of the virus serotypes caused changes in the mRNA expression of this gene (whereas without silencing, the changes were significant, e.g. RQ = 12 for WI‐38). Interesting results were observed in TGF‐β1 mRNA expression (Figure 4B), as no changes were shown after infection with any of the rhinoviruses serotypes used (*p* > 0.05). Nevertheless, in both fibroblast lines ciglitazone decreased HRV‐16‐induced TGF‐β1 mRNA expression and these changes were statistically significant (e.g. RQ = 1.71 for HRV‐16 vs. 0.57 for HRV‐16+ciglitazone in WI‐38 cells, Figure 4B). siRNA knockdown of NF‐κB also abolished ciglitazone‐induced changes for α‐SMA expression (Figure 4E). No statistically significant changes were observed when protein expression was analysed in this set of experiments (*p* > 0.05).

#### 3.2.2 c‐Myc

**FIGURE 5:**
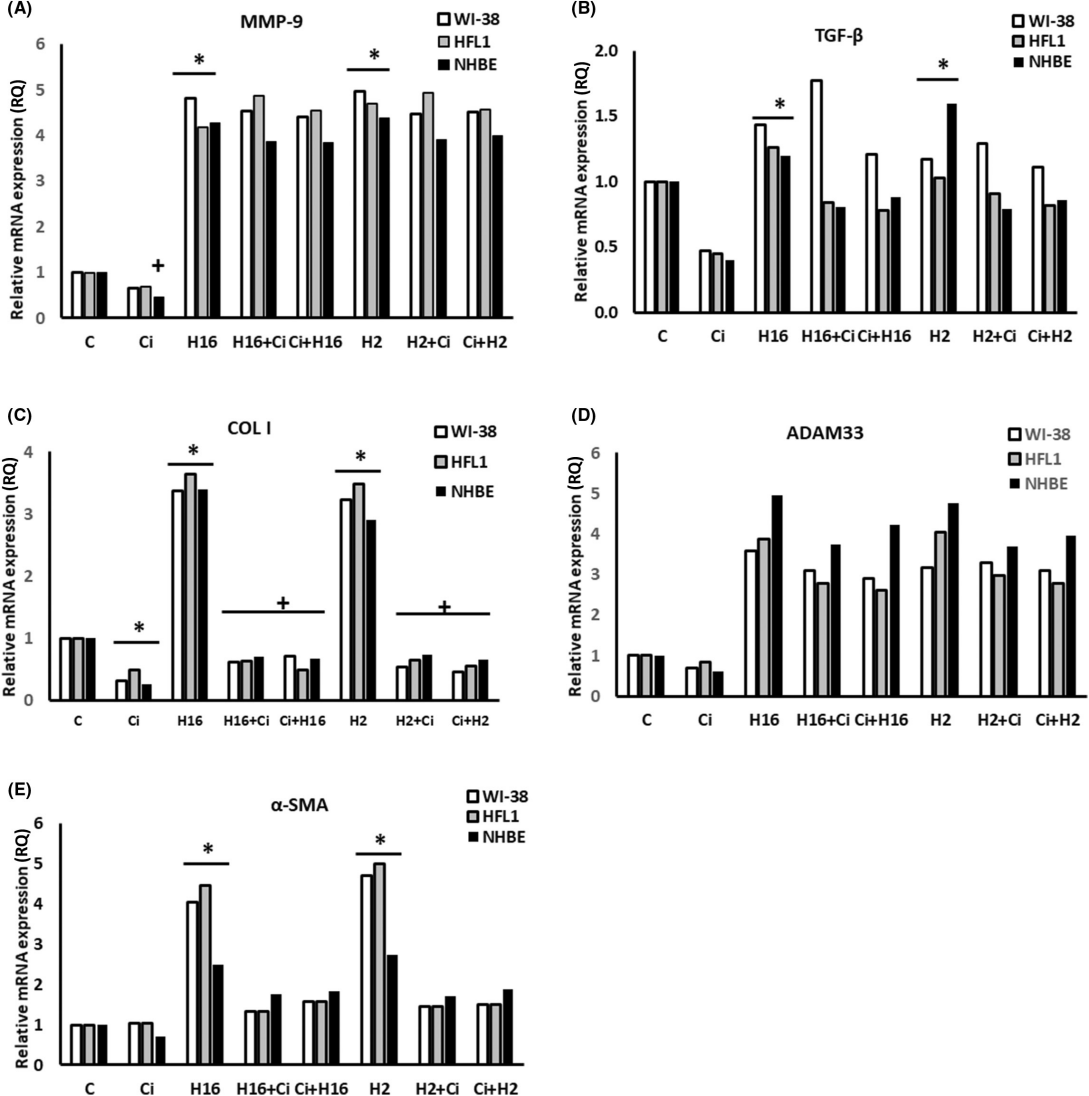
Action of ciglitazone (Ci) in c‐Myc knockdown cells under Rhinovirus infection conditions. Rhinovirus 2 (minor serotype, H2) and 16 (major serotype, H16) were used in the experiments. siRNA silencing of c‐Myc transcription factor reversed the changes in the expression of MMP‐9 (A), TGF‐β (B), COL I (C) ADAM33 (D) and α‐SMA (E). Interestingly, under condition of c‐Myc silencing, ciglitazone effect on COL I was stronger and it significantly decreased HRV‐induced expression of this gene. *p < 0.05 in comparison to the control sample, +*p* < 0.05 in comparison to the Rhinovirus sample (HRV‐2 or HRV‐16, respectively). Ciglitazone (Ci) has been added before rhinovirus infection (Ci+H16, Ci+H2) or after rhinovirus infection (H16+Ci, H2+Ci), C – control sample with medium only.

siRNA silencing of c‐Myc transcription factor affected the mRNA expression of MMP‐9, TGF‐β1, ADAM33 and α‐SMA – no ciglitazone‐induced changes were observed (*p* > 0.05, Figure 5A,B,D,E), although the increase in HRV‐2‐ and HRV‐16‐induced mRNA expression remained unaffected (*p* < 0.05). Knockdown of this transcription factor also abolished HRV‐2‐ and HRV‐16‐induced YKL‐40 mRNA expression changes and the appearance of virus‐induced RXFP1 mRNA expression, but only in epithelial cells (Figure S1H,I, *p* < 0.05).

Under these conditions, ciglitazone induced a statistically significant decrease in COL I mRNA expression in all lines tested, and significantly decreased HRV‐2‐ and HRV‐16‐induced mRNA expression of this gene (Figure 5C, *p* < 0.05).

**FIGURE 6:**
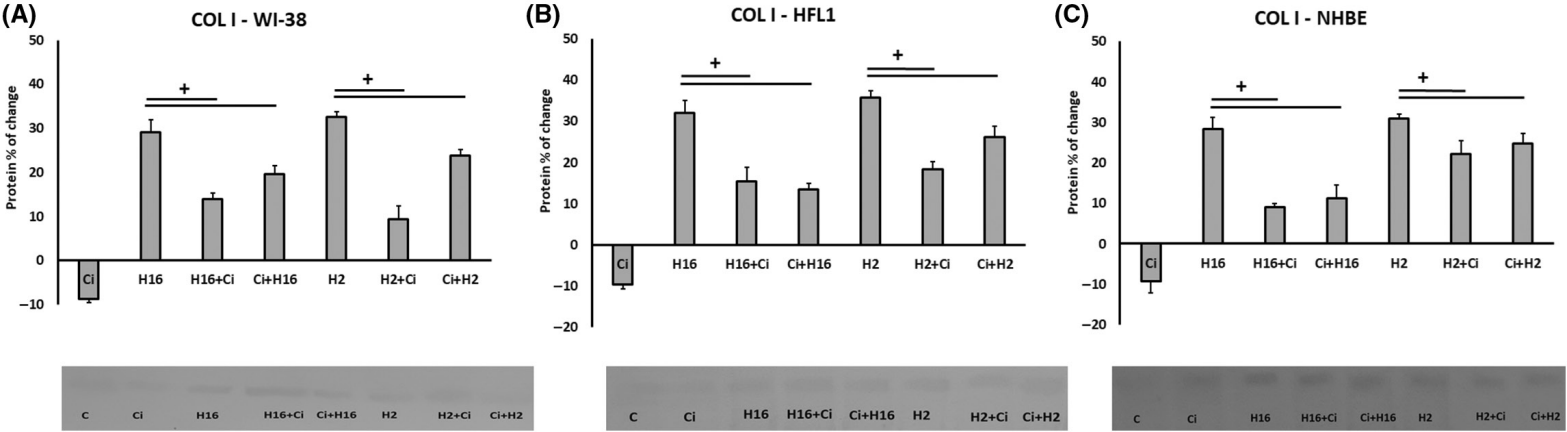
Collagen I protein expression in experimental set with c‐Myc siRNA knockdown. In each cell type – fibroblasts (A, B) and epithelial cells (C), collagen I protein expression was induced by Rhinovirus (both serotypes) and decreased by ciglitazone (Ci) after 24 h incubation. No differences were observed in the order of HRV and CI stimulation. Collagen I concentrations were measured in supernatants utilizing immunoassay in triplicate. Ciglitazone (Ci) has been added before rhinovirus infection (Ci+H16, Ci+H2) or after rhinovirus infection (H16+Ci, H2+Ci). Data presented as % of change, normalizing to control sample ± SD +<0.05.

Protein expression analysis revealed statistically significant effect of ciglitazone on rhinovirus‐induced (both serotypes) expression of collagen I regardless of the order of stimulant addition and in all cell types used in the experiments (Figure 6A–C, *p* < 0.05). The expression of other proteins showed no statistical significance (*p* > 0.05).

#### 3.2.3 STAT3

**FIGURE 7:**
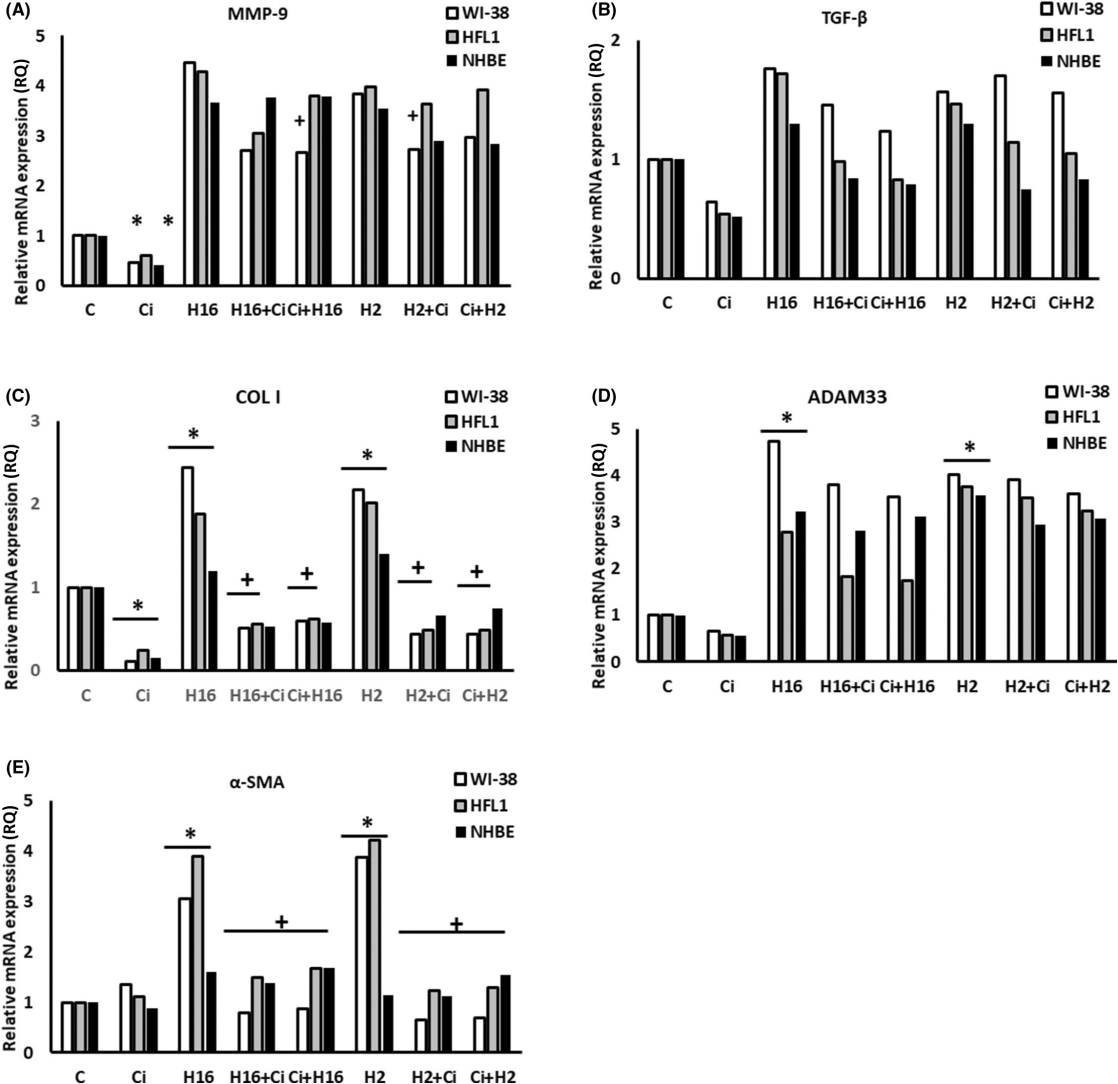
Effects of STAT3 knockdown on ciglitazone (Ci) abilities under Rhinovirus infection conditions. Rhinovirus 16 (H16 in the Figure, major serotype) and 2 (H2 in the Figure, minor serotype) and were used. In comparison to the conditions without the knockdown, the MMP‐9 (A) and TGF‐β (B) mRNA expressions were decreased. Ciglitazone effect were observed in COL mRNA expression, especially its decreasing effect on HRV‐induced COL and a‐SMA expression (C, E). No effect of ciglitazone was observed in ADAM33 (D). *p < 0.05 in comparison to the control sample, +*p* < 0.05 in comparison to the Rhinovirus sample (HRV‐2 or HRV‐16, respectively). Ciglitazone (Ci) has been added before rhinovirus infection (Ci+H16, Ci+H2) or after rhinovirus infection (H16+Ci, H2+Ci). C – control sample with medium only.

STAT3 knockdown resulted in reduced RQ values for MMP‐9 mRNA expression after rhinovirus infection (Figure 7A, e.g. RQ = 4.47 and 4.28 for WI‐38 and HFL1 at HRV‐16, respectively, compared to RQ = 12.61 and 13.29 without silencing). Despite this, ciglitazone under these conditions, caused a reduction in rhinovirus‐induced (both serotypes), MMP‐9 mRNA expression (Figure 7A). For TGF‐β1, RQ values for all samples tested were close to 1, with no statistically significant changes shown (Figure 7B). The results obtained for COL I under STAT3 silencing conditions showed statistically significant reduction in the mRNA expression of this gene under the influence of ciglitazone. This stimulant also decreased HRV‐2‐and HRV‐16‐induced COL I gene mRNA expression (Figure 7C, *p* < 0.05). The mRNA expression of the gene for LTC4 synthase, which played a role of inflammation marker in this study, was significantly reduced by ciglitazone (*p* < 0.05), moreover, in the HFL1 line, a reduction in rhinovirus‐induced (both serotypes) mRNA expression of this gene was observed (*p* < 0.05, Figure S1L).

**FIGURE 8:**
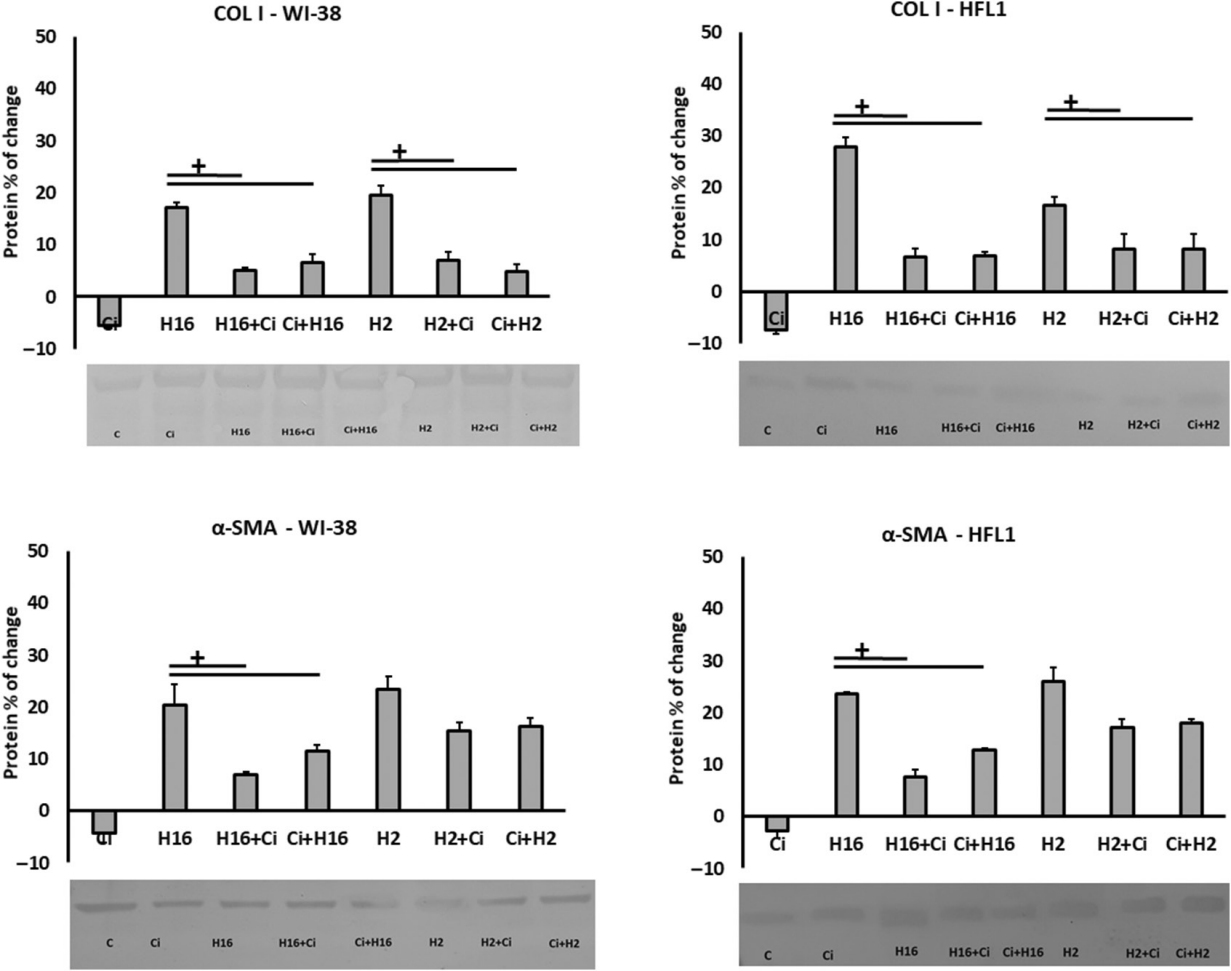
The result of siRNA knockdown of STAT3 on collagen I and α‐SMA protein expression. Ciglitazone (Ci) has been added before rhinovirus infection (Ci+H16, Ci+H2) or after rhinovirus infection (H16+Ci, H2+Ci). The confirmation of mRNA expression on protein level was observed in COL I (A, B) and α‐SMA (C, D). Ciglitazone (Ci) reversed the HRV‐induced augmentation, regardless the virus serotype and the order of stimulation. α‐SMA protein expression has been decreased significantly only after HRV‐16 (H16) stimulation (*p* < 0.05). The bands presented were obtained from Western Blot analysis. The concentrations were measured in supernatants utilizing immunoassay in triplicate. Data presented as % of change, normalizing to control sample ± SD. +<0.05.

Protein expression analysis showed statistical significance in both WI‐38 and HFL1 fibroblast lines for COL I (Figure 8A,B). Rhinovirus‐induced (HRV‐2 and HRV‐16) expression of this protein was reduced by ciglitazone, regardless of stimulation order (*p* < 0.05). Similar results were obtained in the case of α‐SMA, however, only in samples treated with HRV‐16 (Figure 8C,D). No other statistically significant changes were observed when protein expression was analysed in this set of experiments (*p* > 0.05).

**FIGURE 9:**
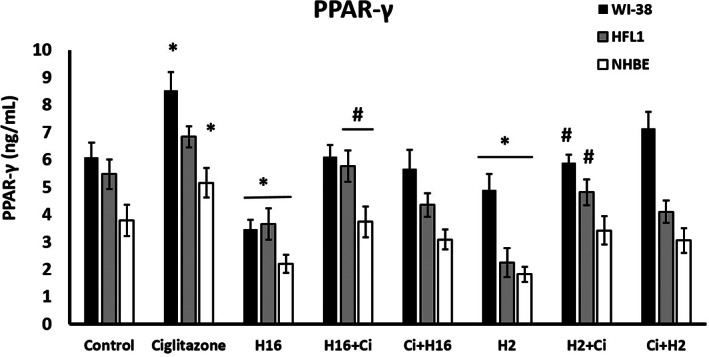
Effects of ciglitazone and two Rhinovirus serotypes on PPAR‐ levels in fibroblast and epithelial cells. Ciglitazone caused an increase in PPAR‐γ while both rhinovirus serotypes decreased it. Ciglitazone added after HRV infection increased PPAR‐γ concentrations in HFL1 fibroblasts and epithelial cells. Ciglitazone (Ci) has been added before rhinovirus infection (Ci+H16, Ci+H2) or after rhinovirus infection (H16+Ci, H2+Ci). Data presented as mean ± SEM, *p < 0.05 in comparison to control, #*p* < 0.05 in comparison to HRV16 or HRV2.

Additionally, the evaluation of PPAR‐γ concentrations in all the cell lines used (Figure 9). The data showed that PPAR‐ is reduced under the influence of rhinovirus, but ciglitazone w can modulate these concentration changes.

## 4 DISCUSSION

Airway inflammation and remodelling are the two characterized pathological features of asthma. PPAR‐γ exerts significant influence on inflammatory responses through acting on T cells, macrophages, dendritic cells, and mast cells, as indicated by the literature.^26^, ^27^, ^28^ When activated, PPAR‐γ in smooth muscle cells exhibit anti‐inflammatory effects as opposed to corticosteroid effects.^29^ PPAR‐γ agonists suppress the production of pro‐inflammatory cytokines such IL‐1, TNF‐α, and IL‐6 in monocytes and macrophages.^30^ Taking into consideration the anti‐inflammatory potential of PPAR‐γ, we aimed to evaluate its agonist – ciglitazone in airway remodelling phenomenon and estimate the roles of chosen transcription factors as possible elements of the pathways.

Consistent with the literature, and our previous studies, human rhinovirus increased the expressions of the genes involved in airway remodelling. This is connected with the fact that HRV is one of the cause of exacerbations and development of airway remodelling.^31^, ^32^

In our study, ciglitazone decreased expression of TGF‐β and MMP‐9 expression – the genes important in fibrosis and thus, crucial in airway remodelling. Interestingly, ciglitazone reversed rhinovirus effect – TGF‐β and MMP‐9 expression in both, fibroblasts and epithelial cells decreased after stimulation.

In our study, the effect of ciglitazone in two aspects was analized – when added to cells before HRV infection and also, when added to already‐infected cells. The order of stimulation may have affected its ciglitazone action and downstream pathways; nevertheless, we showed no significant differences between the results in terms of the order in which ciglitazone was added. Although there are no reports in the literature analysing the effect of ciglitazone in the presence of rhinovirus, the fact that HRV is an inducer of airway remodelling makes the effect on ciglitazone, regardless of the order of addition, potentially clinically relevant. In asthma condition, as shown by the large amount of evidence, MMP‐9 levels are greater in asthmatics than in controls and in severe persistent asthma than in moderate persistent asthma. Moreover, higher concentrations of MMP‐9 are related to lower lung functions. The effects of ciglitazone presented here might be an effect of suppression of the transcription factor NF‐κB activity is in line with earlier findings that suggested PPAR‐γ linked to the inhibition of MMP‐9 transcription. It is still unclear how PPAR‐γ activity reduces MMP‐9 transcription, although studies have shown that PPAR‐γ does not bind to the MMP‐9 promoter directly. Instead, activation of PPARγ‐ represses MMP‐9 production, at least in part, through inhibiting the NF‐κB signalling pathway. In fact, it was discovered that PPAR‐γ activation by ox‐LDL led to considerably less NF‐κB binding to the κB site, moreover, LXR/RXR heterodimers were proved to inhibit expression of MMP‐9 gene in a similar manner.^33^ As PPARγ inhibits the oxidative radicals, it may be also assumed that MMP‐9 expression is also dependent on ROS. Walter et al demontrated recently, that MMP‐9 expression correlates with the reduced ROS levels. Previous studies reported such dependence in cancer cells and astrocytes.^34^, ^35^

TGF‐β and growth factors, among other inflammatory cytokines, are essential for the synthesis and release of MMP‐9.^36^ TGF‐β is a profibrotic cytokine, its isoforms are implicated in the changes of ECM observed during fibrosis. TGF‐β expression is increased in asthmatic airways and appears to correlate with disease severity and degree of subepithelial fibrosis.^37^ In vitro, TGF‐β is able to induce secretion of a numerous extracellular matrix proteins, e.g. collagen I and III, proteoglycans and fibronectin, in fibroblasts.^15^, ^38^, ^39^

It is also a essential cytokine for the induction of collagens after tissue injury. It is known that that collagen I, is the most abundant protein of extracellular matrix, deposited in the asthmatic airways and a hallmark of fibrosis.^40^ In our study, collagen expression increased upon rhinovirus stimulation, however ciglitazone hampered this action in epithelial cells but not in fibroblasts. This is quite unexpected as fibroblasts are the major players in the production of collagen I during fibrosis. We thus hypothesize that this might be due to the low ciglitazone concentration, or the limitation resulting from monoculture of fibroblasts and epithelial cells. Current knowledge recognizes airway fibroblasts and epithelial cells as the two cell types fundamentally engaged in the processes leading to tissue healing following damage. Their close contacts with other parenchymal and/or inflammatory cells appear to enhance their significant metabolic and immunologic features.^41^

Furthermore, collagen gene expression in fibroblasts is suppressed by PPAR‐γ receptor agonists. Effective inhibitors of TGF‐β1‐induced collagen synthesis and COL1A2 gene promoter activity include 15d‐PGJ2 and thiazolidinedione. Additionally, dominant‐negative PPAR‐γ expression vectors prevent 15d‐PGJ2 from inhibiting TGF‐β stimulated COL1A2 promoter activity.^18^

Next to collagen I, α‐SMA is widely used as indicator of fibrosis, including lung fibrosis. In this study it was used as marker of myofibroblast differentiation. The expression pattern of α‐SM actin in cells has been related to a transition from proliferative to differentiated state and cell–cell contact or communication.^42^ Previous chinese study demonstrated that inhibition of alpha‐SMA expression alleviate airway remodelling in mice.^43^ We demonstrate that ciglitazone dicrease rhinovirus‐induced α‐SMA expression, thus showing possible potential of this drug. Surprisingly, the expression of α‐SMA appeared only in fibroblasts, after preincubaiton with ciglitazone, however, regarding fibroblasts, these data are consistent with the literature – TGF‐β1 has been shown to induce ECM and α‐SMA expression by activating the PI3 kinase/Akt pathway and PPAR‐γ agonists suppress TGF‐β1‐induced synthesis of ECM and α‐SMA by blocking Akt phosphorylation.^44^ They are also internally consistent, as ciglitazone simultaneously reduced the expression of the studied genes closely involved in airway remodelling but also influencing each other in this process. It is, however important to consider the data with caution, as previous studies have shown^44^ that the anti‐fibrogenic effect of PPAR‐γ agonists can be mediated through both PPAR‐γ‐dependent^45^ and PPAR‐γ‐independent pathways.^46^, ^47^ This issue was not analysed in the study.

Asthma and bronchial hyperresponsiveness (BHR) are susceptibility conditions linked to the disintegrin and metalloprotease 33 (ADAM33) gene. Studies show that soluble ADAM33 causes structural remodelling of the airways; ADAM33‐induced airway remodelling is reversible.^48^ Furthermore, in a rat model of allergic asthma, silencing of ADAM33 decreased the proliferation and increased apoptosis.^49^ Consistent with these observations, our results have shown that ADAM 33 expression is decreased by ciglitazone, nevertheless, these observations occurred in fibroblasts, not in epithelial cells. It is plausible that ADAM33 mediates the airway inflammation and remodelling brought on by environmental exposure, which may happen through activating TGF‐β signalling and a number of key receptors (CLRs, AhR, and TLRs). Consequently, ADAM33 is a possible asthma target. According to some reports, ADAM33 may have an impact on the epithelial‐mesenchymal trophic unit (EMTU).^50^ Additionally, the soluble form of ADAM33 rapidly induces endothelial cell differentiation in vitro as well as ex vivo and in vivo neovascularization, indicating that ADAM33 might encourage angiogenesis and result in airway remodelling.^51^

In order to evaluate potential signalling pathways in remodelling process in context of rhinovirus infections and stimulation of ciglitazone, we inhibited NF‐κB, c‐myc and STAT 3 transcription factors.

Most inflammatory responses activate NF‐κB through a number of interconnected mechanisms. Some nuclear receptors, including the peroxisome proliferator‐activated receptors, have been identified as some of the numerous regulators influencing these signalling cascades.^52^ In our study knockdown of NF‐κB reversed ciglitazone effects regarding the expression of MMP‐9, COL I, α‐SMA and significantly reduced the results of this stimulant on TGF‐β and ADAM 33 expression. NF‐κB appears to be an important agent in the expression of airway remodelling‐ related genes – according to the literature, it participates in the expression of the genes mentioned.^53^, ^54^, ^55^ Nevertheless, the study failed to show the influence of ciglitazone on the evaluated genes in such conditions.

Similar results were obtained in our study after c‐Myc siRNA silencing – reduction of TGF‐β and inversion of MMP‐9, ADAM33 and α‐SMA expression. It has been demonstrated that the promoter regions of several inflammatory genes (IL‐6 and TNF‐α) physically connect with the regulatory transcription factor c‐Myc, which controls cell proliferation. Little is known regarding a role of this transcription factor in airway remodelling. Recently, Shen et al^56^ demonstrated that by stimulating TGF‐β signalling, it contributes to the stimulation of fibroblasts and subsequent synthesis of ECM proteins. in mouse model renal fibrosis. This work partially confirms our results, showing that c‐Myc knockdown, inhibits expression and gathering of fibronectin, collagen I, and β‐SMA among other ECM proteins. Interestingly, we illustrated here unexpected result of c‐Myc silencing – in our study collagen I expression was significantly decreased by ciglitazone, both alone and under conditions of rhinovirus infections. A previouss work more thematically distant, revealed connection of c‐Myc with impaired healing, a rise in c‐myc expression and a decline in the collagen I/III ratios.^57^ We hypothesize that collagen I may have more than one expression pathways which may be swiched, however, it is difficult to support it, as no data is available regarding this issue.

siRNA inhibition of STAT3 transcription factor, reversed the changes in TGF‐β expression caused by ciglitazone and rhinoviruses, it also significantly reduced the expression of ADAM33 expression, which might mean that this transcription factor is pivotal for the expression of those genes.

Collagen I expression RQ values have been reduced, nevertheless, it was detectable. Moreover, ciglitazone significantly reduced COL I expression, however only in fibroblasts. There are opposite data available – Papaioannou et al^58^ suggested that STAT3 is a strong enhancer of COL I expression, nevertheless numerous well‐known transcription factors have been linked to basal collagen gene expression (e.g. Sp1, AP1, Smads, or c‐Krox) and have been found to interact with the promoters and upstream regulatory regions of collagen I genes.^59^ This issue requires further research, however, it seems interesting what pathways determine switch to appropriate transcription factor in collagen I transcription process under different conditions.

Our study has some limitation that we are aware of – the cell culture model does not fully reflect the nature of the cells during airway remodelling process, so the data presented should be interpreted with caution. In addition, the lines are monocultures, so the effect of the two cell types on each other was limited. Additionally, thiazolidinediones have been withdrawn from the clinical use in diabetes due to the possible cardiovascular complications. Nevertheless, this group of drugs are still of research value because of their property of activating PPARγ.^60^

## 5 CONCLUSION

In conclusion, in this study we found that ciglitazone stimulation attenuated expression of some genes strongly connected to airway remodelling. Moreover, ciglitazone decreased rhinovirus‐induced expression of the most important fibrotic genes – MMP‐9, TGF‐β and collagen I suggesting that the therapeutic effect on airway remodelling exerted by ciglitazone, was associated with activation of PPARγ and its down‐stream pathways. In this process, NF‐κB, c‐Myc and STAT3 transcription factors may contribute. Further investigation into the mechanisms through which PPAR‐γ inhibits airway remodelling are needed.

## AUTHOR CONTRIBUTIONS

**Joanna Wieczfinska:** Conceptualization (equal); data curation (lead); formal analysis (lead); funding acquisition (lead); investigation (lead); methodology (lead); project administration (lead); supervision (supporting); visualization (supporting); writing – original draft (lead). **Rafal Pawliczak:** Formal analysis (supporting); supervision (lead); writing – review and editing (lead).

## FUNDING INFORMATION

This work was supported by a grants from the National Science Centre, Poland No. 2015/19/D/NZ6/02988 and by Medical University of Lodz, No. 503/0‐149‐03/503‐01‐001‐19‐00.

## CONFLICT OF INTEREST STATEMENT

The authors have no relevant financial or non‐financial interests to disclose.

## Supporting information


Figure S1.

## Data Availability

The data presented in this study are available on request from the corresponding author. The data are not publicly available due to privacy restrictions.
