# Supplementary material for: Anti‐fibrotic effect of ciglitazone in HRV‐induced airway remodelling cell model
Source: J Cell Mol Med. 2023 May 31;27(13):1867–79. doi: 10.1111/jcmm.17790 (PMC10315786; doi:10.1111/jcmm.17790)
Supplement: Supplementary file 1 — Figure S1. [file JCMM-27-1867-s001.docx]

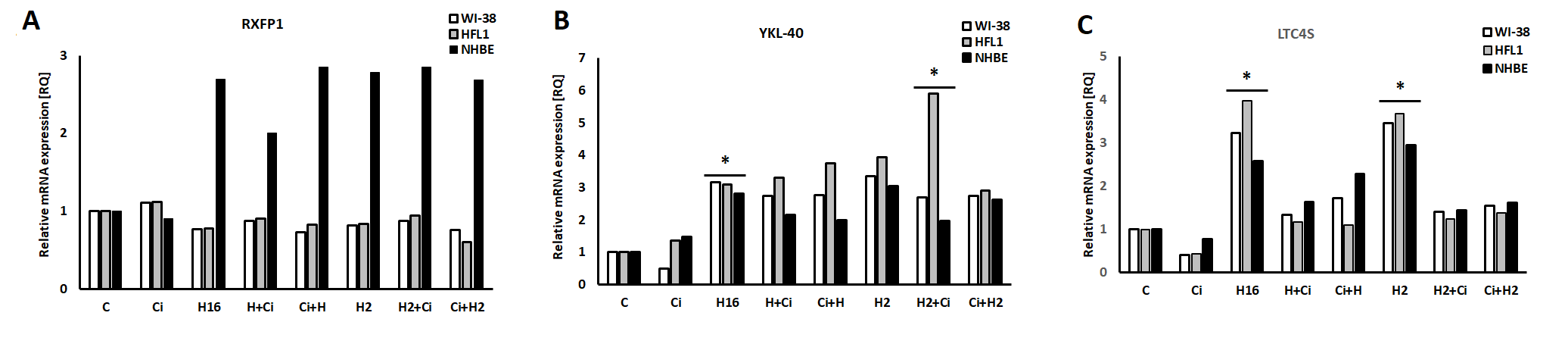


siRNA NF-κB


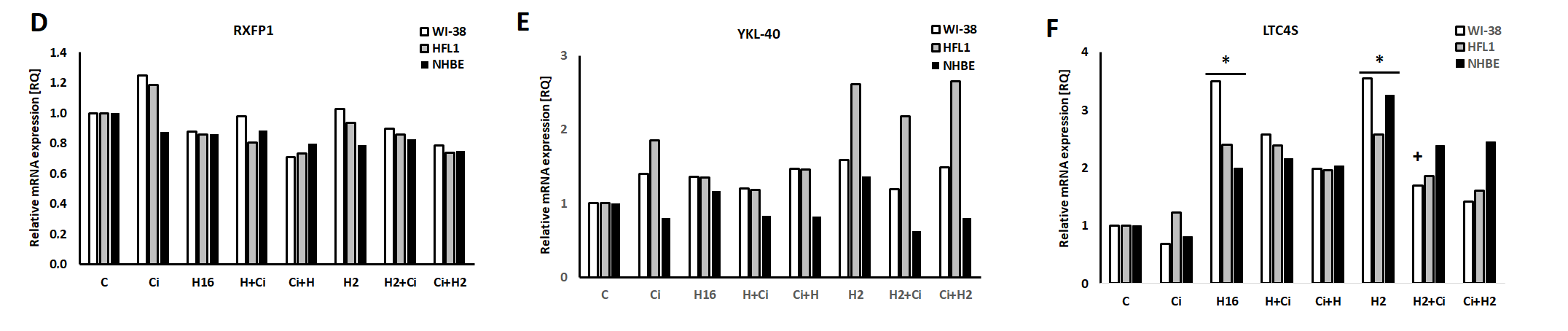


siRNA c-Myc


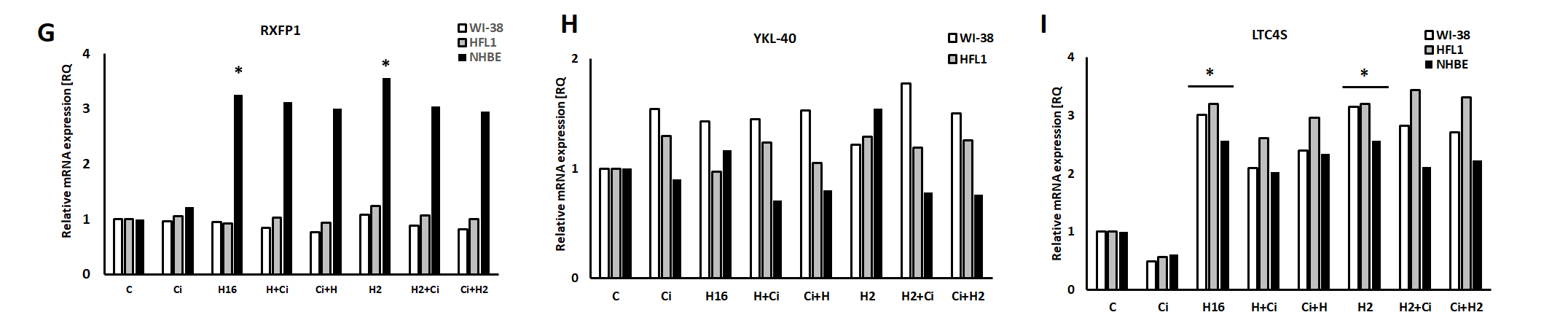


siRNA STAT3


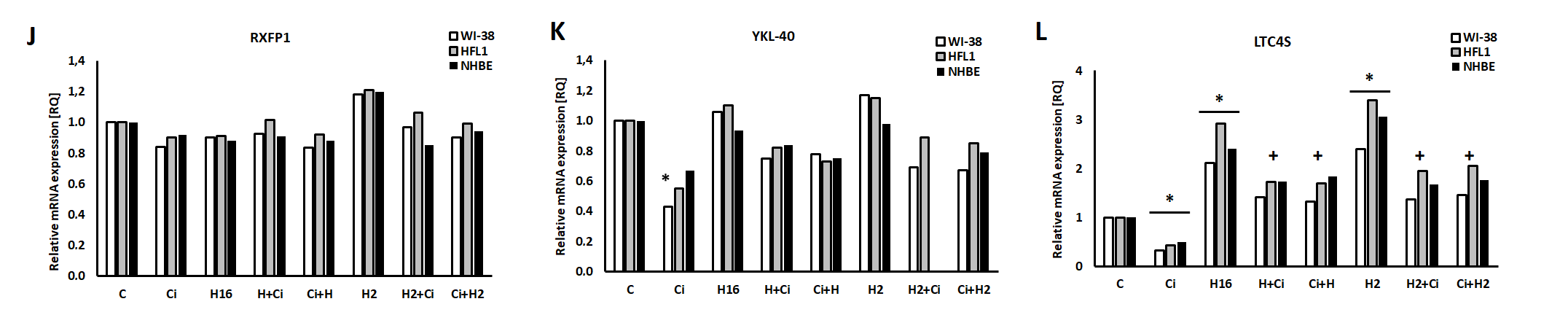


**Figure S1.** Data Presenting RXFP1 (A, D, G, J), YKL-40 (B, E, H, K) and LTC4 (C, F, I, L) mRNA expression. The cells were stimulated with ciglitazone (Ci) and 2 serotypes of human rhinovirus (HRV-2, H2 and HRV-16, H16). Rhinovirus induced the mRNA expression of LTC4S, which was used in the study as the inflammation marker. Only in HFL1 fibroblast cell line we observed the effect of ciglitazone in HRV-induced mRNA expression. YKL-40 which acts as a soluble mediator of cell proliferation and migration, has not been changed in our study – by ciglitazone. Nevertheless, mRNA expression of this gene was induced in cells but not under the condition of silencing any of transcription factors used.

Relaxin receptor - RXFP1 (relaxin family peptide receptor (RXFP) mRNA expression did not change significantly in fibroblasts, but only in epithelial cells under the conditions of silenced c-Myc transcription factor. Endogenous relaxin appears to play an important role in regulating collagen deposition within the lung and published data further confirmed the potential role of exogenous relaxin in the fibrosis associated with airway and lung disorders, however, these results do not confirm ciglitazone influence on RXFP1 expression.
